# Basophil Activation-Dependent Autoantibody and Interleukin-17 Production Exacerbate Systemic Lupus Erythematosus

**DOI:** 10.3389/fimmu.2017.00348

**Published:** 2017-03-27

**Authors:** Qingjun Pan, Li Gong, Haiyan Xiao, Yongmin Feng, Lu Li, Zhenzhen Deng, Ling Ye, Jian Zheng, Carol A. Dickerson, Lin Ye, Ning An, Chen Yang, Hua-feng Liu

**Affiliations:** ^1^Key Laboratory of Prevention and Management of Chronic Kidney Disease of Zhanjiang City, Affiliated Hospital of Guangdong Medical University, Zhanjiang, China; ^2^Department of Laboratory Animal Center, Nanfang Hospital, Southern Medical University, Guangzhou, China; ^3^Department of Anesthesiology and Perioperative Medicine, Medical College of Georgia, Augusta University, Augusta, GA, USA; ^4^Department of Microbiology, University of Iowa, Iowa City, IA, USA

**Keywords:** basophil, IgE, systemic lupus erythematosus, autoantibody, interleukin-17

## Abstract

**Objective:**

Autoantibody and inflammatory cytokines play crucial roles in the development of systemic lupus erythematosus (SLE); however, the regulation of their production warrants further investigation. This study aimed to investigate the role of basophil activation in the development of SLE based on studies in patients with SLE and spontaneous lupus-prone MRL-*lpr/lpr* mice.

**Methods:**

The phenotypes of peripheral basophils and the production of autoantibody and interleukin (IL)-17 in patients with SLE were determined by flow cytometry and enzyme-linked immunosorbent assay, and also their correlations were investigated by statistical analysis. Thereafter, the effect of basophils on autoantibody production by B cells and Th17 differentiation in SLE were evaluated *in vitro*. Finally, the effect of basophil depletion on the development of autoimmune disorders in spontaneous lupus-prone MRL-*lpr/lpr* mice was examined.

**Results:**

The decreased numbers and an increased activation of peripheral basophils were found to be correlated with increased autoantibody production and disease activity in patients with SLE. Correspondingly, *in vitro* coculture studies showed that basophils obtained from patients with SLE promoted autoantibody production by SLE B cells and promoted Th17 differentiation from SLE naïve CD4^+^ T cells. The decrease of peripheral basophils in patients with SLE might be due to their migration to lymph nodes post their activation mediated by (autoreactive) IgE as supported by their increased CD62L and CCR7 expressions and accumulation in the lymph nodes of MRL-*lpr/lpr* mice. Furthermore, an increased activation of peripheral basophils was identified in MRL-*lpr/lpr* mice. Importantly, basophil-depleted MRL-*lpr/lpr* mice exhibited an extended life span, improved renal function, and lower serum levels of autoantibodies and IL-17, while basophil-adoptive-transferred mice exhibited the opposite results.

**Conclusion:**

These finding suggest that basophil activation-dependent autoantibody and IL-17 production may constitute a critical pathogenic mechanism in SLE.

## Introduction

Systemic lupus erythematosus (SLE) is a systemic autoimmune disease that is characterized by the production of a wide spectrum of autoantibodies and inflammatory cytokines. Several types of immune cells are considered critical players in the induction of autoantibodies and inflammatory cytokine production in the pathogenesis of SLE, including overactivated B cells, abnormally activated T cell subsets, monocytes, and dendritic cells (1–3). Of these cell types, overactivated B cells play a central role by directly producing a large quantity of autoantibodies that can lead to systemic inflammation and organ damage (1, 4). However, the regulation of B cell autoantibody production is complex, and a variety of factors, such as other immune cells, environmental triggers, and genetic susceptibility, are involved (1, 5). For abnormally activated T cell subsets, increasing evidence suggests that Th17 cells play a pivotal deleterious role in the inflammation and organ damage that occur in SLE (6, 7); thus, the regulation of Th17 differentiation in SLE warrants further investigation.

T cell-dependent B cell activation has been well characterized; however, T cell-independent B cell activation is not fully understood, particularly in autoimmune diseases (8). Basophils, one of the least abundant populations of granulocytes, are well known to be involved in allergic responses and also play critical roles in acquired immunity regulation and immunological disorders by releasing different patterns of immune modulators such as cytokines and chemokines upon activation by different stimuli (9, 10). It has been confirmed that basophils can deliver helper signals to B cells and drive their differentiation toward antibody-producing cells (11); however, the influence of basophils on autoantibody production by B cells in SLE is largely unknown.

Moreover, whether basophils promote the differentiation of Th17 cells remains controversial (12–15). Differences in the microenvironment and stimulatory conditions could be possible reasons for previous discrepant results. Th17 cells’ development requires synergistic effects mediated by series of cytokines, such as interleukin (IL)-23, IL-1β, TGF-β, and another key cytokine, IL-6, to drive their differentiation (16, 17). It is well documented that murine basophils can express IL-6 under specific conditions (18–20), but the effects of basophils on Th17 differentiation remain largely unknown in the context of SLE.

In 1990s, Hibbs et al. established a Src-family protein tyrosine kinase Lyn-deficient mouse model, which develops strong, constitutive Th2 skewing in early life and exhibits symptoms of an autoimmune disease that mimics some of the features of human SLE in later life (21). Although this model is unlikely to model SLE in the majority of affected persons (22), basophils were shown to be indispensable for the development of autoimmune disease in these Lyn-deficient mice (23), which have provided the possible pathogenesis of basophils in SLE. However, the mechanistic links between basophils activation and SLE, especially its activation on autoantibody and inflammatory cytokines production in SLE, remain to be further elucidated.

This study aimed to investigate the role of basophil activation in the development of SLE based on studies in patients with SLE and spontaneous lupus-prone MRL-*lpr/lpr* mice, with a special focus on the effect of basophil activation on autoantibodies and inflammatory cytokine production in SLE.

## Materials and Methods

### Patients

A total of 126 patients with SLE (107 females and 19 males) (Table 1) and 48 healthy controls (36 females and 12 males) with no differences with regard to age, sex, or race were enrolled into the present study at the Department of Nephrology at the Affiliated Hospital of Guangdong Medical University from October 2012 to October 2015. Forty-eight newly diagnosed patients with active SLE without treatment (here termed newly diagnosed SLE) (Table 1) from the 126 patients with SLE enrolled were the main cohort studied. Fifteen patients (Table 1) were followed up during a 3-month period of treatment. All patients fulfilled the SLE classification criteria of the American College of Rheumatology (Atlanta, GA, USA) (24). The disease activity of the patients with SLE was evaluated using the SLE disease activity index (SLEDAI) (25). Exclusion criteria were as follows: patients with coinfections, allergies, other serious systemic diseases, and other autoimmune disorders.

**Table 1:** **Demographic characteristics of SLE patients**.

	Total SLE patients (*n* = 126)		Newly diagnosed SLE patients (*n* = 48)	Followed-up SLE patients (*n* = 15)	
Age (mean ± SD)	28.7 ± 11.3		24.3 ± 10.5	25.9 ± 10.1	
Gender [F/M, no. (%)]	107 (84.9%)/19 (15.1%)		45 (93.8%)/3 (6.2%)	14 (93.3%)/1 (0.7%)	
Disease duration (mean ± SD, years)	4.7 ± 3.2				
Anti-dsDNA IgG positive: no. (%)	94 (74.6%)		43 (89.6%)	14 (93.3%)	
SLEDAI score					
Mean ± SD	12.2 ± 7.0		17.2 ± 5.2	18.0 ± 5.5	
Median (minimum, maximum)	12 (0, 30)		16 (11, 30)	19 (11, 27)	
Treatment	Treatment	*n* (%)		Patients no. + treatment	
	P	67 (53.2)		1. P + CTX	2. P + MMF
	HCQ	32 (25.4)		3. P + AZA + TW	4. P + TW
	MMF	4 (3.2)		5. P	6. P + CTX + TW
	CTX	61 (48.4)		7. P + CTX	8. P + CTX + HCQ
	AZA	7 (5.6)		9. P + CTX + TW	10. P + CTX
	TW	54 (42.9)		11. P + CTX	12. P + HCQ
				13. P + MMF	14. P + HCQ
				15. P + MMF	

*SLE, systemic lupus erythematosus; SLEDAI, SLE disease activity index; P, prednisone; HCQ, hydroxychloroquine; MMF, mycophenolate mofetil; CTX, cyclophosphamide; AZA, azathioprine; TW, Tripterygium wilfordi*.

This study was approved by the Ethics Committee of the Affiliated Hospital of Guangdong Medical University, and written informed consent was received from each subject.

### Mice

Female MRL-*lpr/lpr* mice were purchased from the Jackson Laboratory (Bar Harbor, ME, USA) and maintained in the pathogen-free facility of the Laboratory Animal Center of Southern Hospital with the approval of the Ethics Committee for Experimental Animals at Nanfang Hospital, Southern Medical University. All experiments were performed according to the national guidelines for animal welfare.

### Flow Cytometric Analysis

Human basophils were gated on FcεRIα-FITC/CD123-PerCP/Cy5.5/CD203c-PE (BioLegend, San Diego, CA, USA) positive cells after extracellular and intracellular staining. The expression levels of CD203c-PE, CD62L-APC, FcεRIα-FITC, CCR7-APC, CD63-APC, IL-13-APC, B cell-activating factor (BAFF)-APC (BioLegend, San Diego, CA, USA), IL-4-PE-Cy7, and IL-6-APC (eBioscience, San Diego, CA, USA) in basophils were quantified and expressed as relative fluorescence units (the ratio of mean fluorescence intensity normalized to controls) or as a positive percentage of total basophils.

Mouse basophils were gated on CD49b-APC/IgE-PE (BioLegend, San Diego, CA, USA). The expression levels of the activation marker CD200R-FITC (BioLegend, San Diego, CA, USA) (26), IL-4-FITC, and IL-6-FITC (eBioscience, San Diego, CA, USA) were quantified and expressed in the same way as for human basophils. A FACScanto™ Π flow cytometer (Becton Dickinson, San Jose, CA, USA) and Lysys II software (Becton Dickinson, San Jose, CA, USA) or FlowJo Software (Tree Star, San Carlos, CA, USA) were used to acquire and analyze the data.

### Basophil Depletion or Adoptive Transfer in MRL-*lpr/lpr* Mice

Depletion of basophils in female MRL-*lpr/lpr* mice was performed by injection of 5 µg anti-mouse FcεRI (MAR1; eBioscience, San Diego, CA, USA) twice daily intraperitoneally for 3 days (18, 27), and control group (non-depleted) mice were treated with an isotype control antibody (eBioscience, San Diego, CA, USA). For basophil-adoptive-transfer, basophils were isolated from the peripheral blood of age-matched MRL-*lpr/lpr* mice using magnetic microbeads against CD49b^+^ (Miltenyi Biotec GmbH, Germany), and FcεRI^+^ and FcεRI^++^ basophils were further isolated by FACS-sorting (Becton Dickinson, San Jose, CA, USA) (27) to yield a purity of greater than 90%. Then, 1 × 10^4^ basophils per mouse were adoptively transferred through the tail vein. The cumulative survival to 36 weeks was monitored. Blood was obtained by puncture of the orbital venous plexus, and serum, urine, lymph nodes, and renal samples were collected at the indicated time points.

### Serum and Urine Analysis

Serum levels of IL-17 in humans and IL-17 and INF-γ in mice were measured using enzyme-linked immunosorbent assay (ELISA) kits (Life Technologies, Grand Island, New York, NY, USA). Serum levels of antinuclear IgG in humans (Zeus Scientific, Inc., Branchburg, NJ, USA) or mice (Cusabio Biotech, Wuhan, China) were measured using ELISA kits. To test for anti-dsDNA IgE in human or antinuclear IgE in mice, an anti-dsDNA IgG ELISA kit (Fuchun Kexin Biotech, Shanghai, China) or an antinuclear IgG ELISA kit (Cusabio Biotech, Wuhan, China) was modified by using HRP-anti-human IgE or HRP-anti-mouse IgE as the secondary antibody, respectively, and the serum samples were diluted at 1:25. The results are expressed as standard units (international units per milliliters, units per milliliters, or microgram per milliliters) or as the absorbance measured at 450 nm following a chromogenic (TMB) reaction (BioLegend, San Diego, CA, USA). Human serum IgE levels were determined using a chemiluminescence immunoassay (Elecsys 2010 analyzer, Roche Diagnostics Ltd., Switzerland).

To test for antinuclear IgE in humans, a line-blot method using the ANA Euroline Profile 3 kit (Euroimmun, Lübeck, Germany) was modified by using AP-anti-human IgE (Thermo Fisher Scientific, San Jose, CA, USA) as the secondary antibody, and the serum samples were diluted at 1:10.

For western blot detection of circulating immune complexes (CICs) containing IgE (IgE-CIC), PEG6000 (Sigma-Aldrich, St. Louis, MO, USA) (3.75%)-precipitated circulating CICs were prepared (28); then, HRP-anti-human IgE (Abcam Inc., Cambridge, MA, USA) was used for detection.

Twenty-four-hour urine was collected from metabolic cages to determine the levels of serum creatinine, C3 (Alpha Diagnostic Intl. Inc., San Antonio, TX, USA), blood urea nitrogen (StressMarq Biosciences Inc., Victoria, BC, Canada), and urinary proteins (Bradford method, Bio-Rad protein assay reagent).

### Tissue Analysis

Immunofluorescence analysis was conducted to measure immunoglobulin deposition in human and mouse kidneys (29). FITC anti-human IgG (Sigma-Aldrich, St. Louis, MO, USA) and human IgE (eBioscience, San Diego, CA, USA), as well as FITC anti-mouse IgG (Santa Cruz Biotechnology, CA, USA) and IgE (eBioscience, San Diego, CA, USA) were employed. For human biopsy, the fluorescence intensity of glomerular staining was graded on a scale from 0 to ++++ in increments of 0.5+. Images were recorded under a TCS SP5 II confocal microscope (Leica Microsystems, Mannheim, Germany).

### Isolation and Activation Assays of Human Basophils

An EasySep™ human basophil enrichment kit (Stemcell Technologies, Vancouver, BC, Canada) was used to negatively purify human basophils to obtain a purity of greater than 90% using flow cytometry (30).

For activation analysis, human basophils isolated from healthy controls were aliquoted at 4,000 cells/well in a total volume of 200 µl medium containing IL-3 (2 ng/ml, PeproTech., London, UK); to these were added the AB serum of healthy controls (20% of a total volume) or newly diagnosed patients with SLE (20% of a total volume), or CICs that were precipitated by PEG 6000 (3.75%) from the serum of newly diagnosed patients with SLE (31), or serum from newly diagnosed SLE patients that flow through a Sepharose 4B™ column (GE Healthcare, Uppsala, Sweden) coupled with anti-IgE antibody to depletion of IgE (32), or anti-IgE antibody (0.5 µg/ml; positive control) (Abcam Inc., Cambridge, MA, USA) at 37°C for 15 min (CD203c detection) or 24 h (CD62L, CCR7, and IL-4-positive detection) (33).

### Autoantibody Production Assay

B cells were isolated from newly diagnosed patients with SLE using an EasySep™ Human B Cell Enrichment kit (Stemcell Technologies, Vancouver, BC, Canada) (34). Then, for the measurement of autoantibody production, B cells (1 × 10^5^ cells/well) were cultured for 12 days with polyclonally activated CD4^+^ T cells (1 × 10^5^ cells/well) from healthy controls using anti-CD3 (0.5 mg/ml) (Miltenyi Biotec GmbH, Germany); or negatively purified basophils (5 × 10^4^ cells/well) from healthy control or newly diagnosed patients with SLE. Cells were cultured in DMEM (300 µl) (10% FBS), IL-2 (10 ng/ml) and IL-3 (10 ng/ml) (PeproTech) (18, 35), as well as nuclear extracts of Hep-2 cells (10 µg/ml) isolated with a Nuclear and Cytoplasmic Extraction Kit (Thermo Fisher) as a stimulator for the first 3 days (36). Levels of antinuclear IgG (Zeus Scientific) and IgE (as described in serum and urine analysis, and culture supernatant samples were diluted 1:4), anti-nucleosomes IgG (ELISA) (Euroimmun, Lübeck, Germany) and IgE, and anti-tetanus IgG (ELISA) (ZhengZhou Etebio Technology Co., Ltd., Zhengzhou, China, approved by SFDA) and IgE in the culture supernatants were measured using ELISA kits.

### Th17 Differentiation Assay

Naïve CD4^+^ T cells from newly diagnosed patients with SLE using an EasySep™ Human Naïve CD4^+^ T Cell Enrichment Kit (Stemcell technologies) were stimulated with anti-CD2/CD3/CD28 T cell activation beads (Miltenyi Biotec) at a bead-to-cell ratio of 1:2 for 3 days, followed by incubation for 4 days in the presence of a Th17 differentiation cytokine mixture (IL-2, 20 ng/ml; IL-6, 20 ng/ml; IL-23, 20 ng/ml; IL-1β, 20 ng/ml; TGF-β1, 5 ng/ml; anti-IL-4, 5 µg/ml; anti-IL-12, 5 µg/ml; anti-IFN-γ. 1 µg/ml; PeproTech., London, UK), or culturing in the Th17 differentiation cytokine mixture (excluding IL-6) with negatively isolated basophils (2 × 10^4^ cells/well) from healthy controls, or from newly diagnosed patients with SLE in the absence or presence of anti-IL-6 (5 µg/ml, PeproTech., London, UK), respectively.

### Statistics

All statistical analysis was performed using SPSS 16.0 (SPSS, Inc., Chicago, IL, USA). Two-group comparisons were performed using unpaired two-tailed Student’s *t*-test. Multiple-group comparisons were performed using one-way analysis of variance (ANOVA) followed by the Bonferroni or Dunnett *post hoc* tests. Survival curves were analyzed using the non-parametric *Kaplan–Meier method*. Spearman’s rank correlation was used to detect correlations among different study parameters. A *P*-value of <0.05 was considered to indicate statistical significance. Each symbol represents an individual patient, one mouse, or one sample. The data are presented as scatter plots and are expressed as means.

## Results

### Decreased Numbers and Increased Activity of Peripheral Basophils Are Correlated with Disease Activity and Increased Autoantibody Production in Patients with SLE

**Figure 1:**
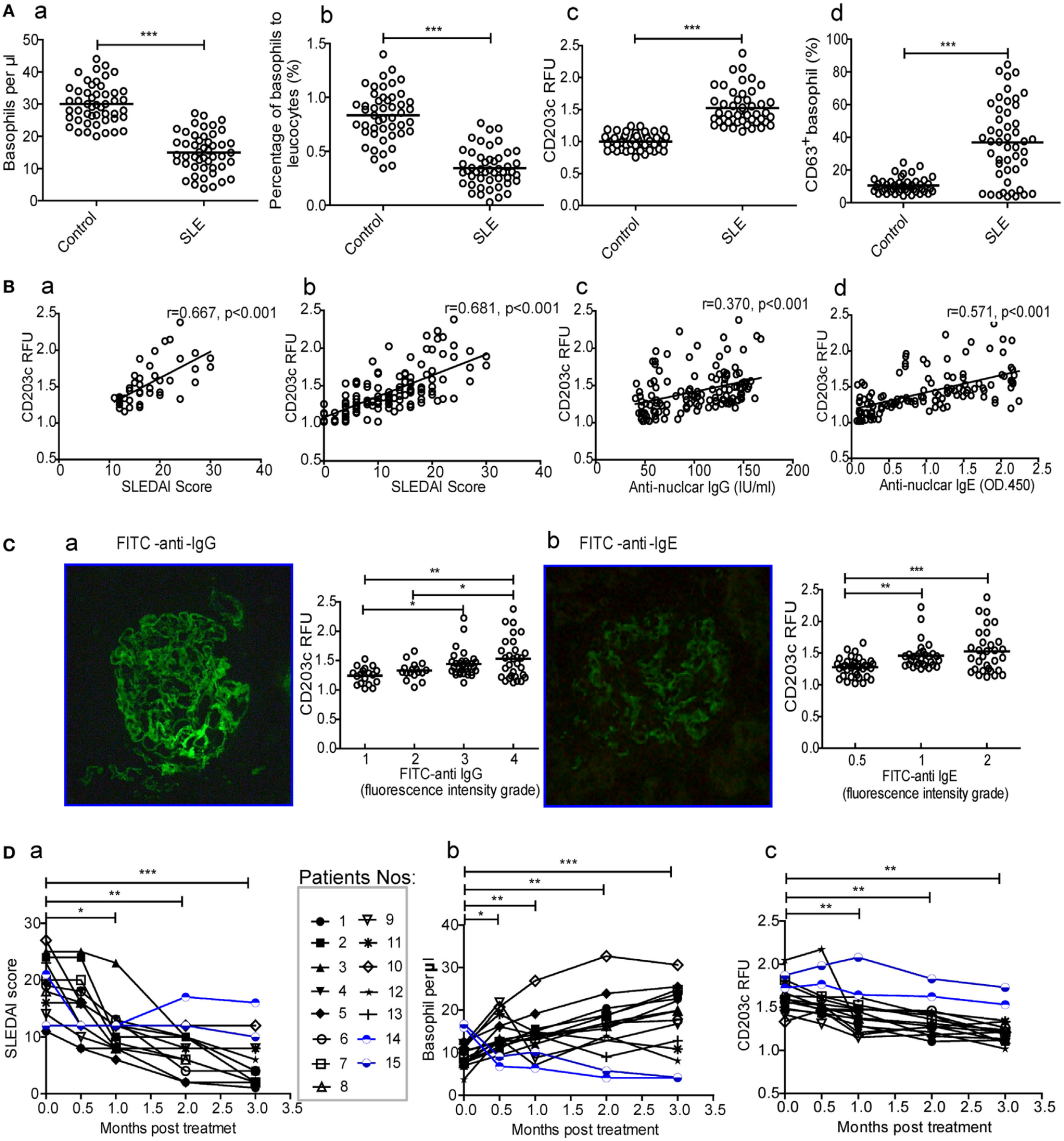
**Numbers and activation of peripheral basophils and their correlations with disease activity and autoantibody production in patients with systemic lupus erythematosus (SLE)**. **(A)** The number (a, b) and activation (CD203c expression and CD63 positive percentage) (c, d) of peripheral basophils in newly diagnosed patients with SLE (*n* = 48) and healthy controls (*n* = 48) were detected using flow cytometry. **(B)** Correlations between the activation (CD203c expression) of peripheral basophils and disease activity [SLE disease activity index (SLEDAI) score] of newly diagnosed patients with SLE (*n* = 48) (a), or total patients with SLE (*n* = 126) (b), levels of their serum antinuclear IgG (*n* = 126) (c), and IgE (*n* = 126) (d) were analyzed. **(C)** The association between the activation (CD203c expression) of peripheral basophils in patients with SLE (*n* = 97) who received renal biopsy and the deposition of IgG (a) and IgE (b) evaluated by fluorescence intensity grade in biopsied kidneys. **(D)** Changes in the SLEDAI score (a), the numbers (b), and activation (CD203c expression) (c) of peripheral basophils in patients with SLE (*n* = 15) who were followed up for 3 months posttreatment. *P < 0.05, **P < 0.01, ***P < 0.001. Data were analyzed *via* Student’s *t*-test **(A,D)**, Spearman’s rank correlation **(B)**, or one-way analysis of variance **(C)** and presented as scatter plots and are expressed as means.

To assess whether basophils play a role in SLE, the numbers and activation of peripheral basophils and their correlations with disease activity (SLEDAI score) were evaluated in patients with SLE. The results showed that the number (Figure 1A—a) and percentage (Figure 1A—b) of peripheral basophils in newly diagnosed patients with SLE were significantly decreased compared with healthy controls. However, these basophils were activated and exhibited higher expression levels of activation makers such as CD203c and CD63 (Figure 1A—c and d) compared with those from healthy controls. The representative data of FACS for basophil frequency, CD203c, and CD63^+^ were also shown (Figure S1 in Supplementary Material) The higher expression of CD203c on basophils correlated with disease activity, as assessed by the SLEDAI score, not only in the newly diagnosed SLE patients (Figure 1B—a) but also in the total group of patients with SLE (Figure 1B—b). Additionally, the higher expression of CD203c on basophils correlated with the serum levels of antinuclear IgG and IgE (Figure 1B—c and d) along with the deposition of IgG and IgE in kidney biopsies (Figure 1C) in the total group of patients with SLE. Furthermore, a follow-up study showed that after treatment with immunosuppressants and other drugs, newly diagnosed patients with SLE who experienced effective treatment (defined as a decrease in the SLEDAI score of more than 6 and the absence of damage to the heart, lungs, brain, blood, intestines, or any other vital organs) showed an increased level of peripheral basophils and decreased basophil activity as indicated by the expression of CD203c (Figure 1D, nos.1–13); in contrast, patients who did not experience effective treatment did not show any of these signs (Figure 1D, nos.14 and 15). Also, patients with SLE who treated with *Tripterygium wilfordi* have been shown apart (Figure S2 in Supplementary Material). These findings indicate that peripheral basophil activation may play an important role in SLE.

### (Autoreactive) IgE-Mediated Basophil Activation in Patients with SLE

**Figure 2:**
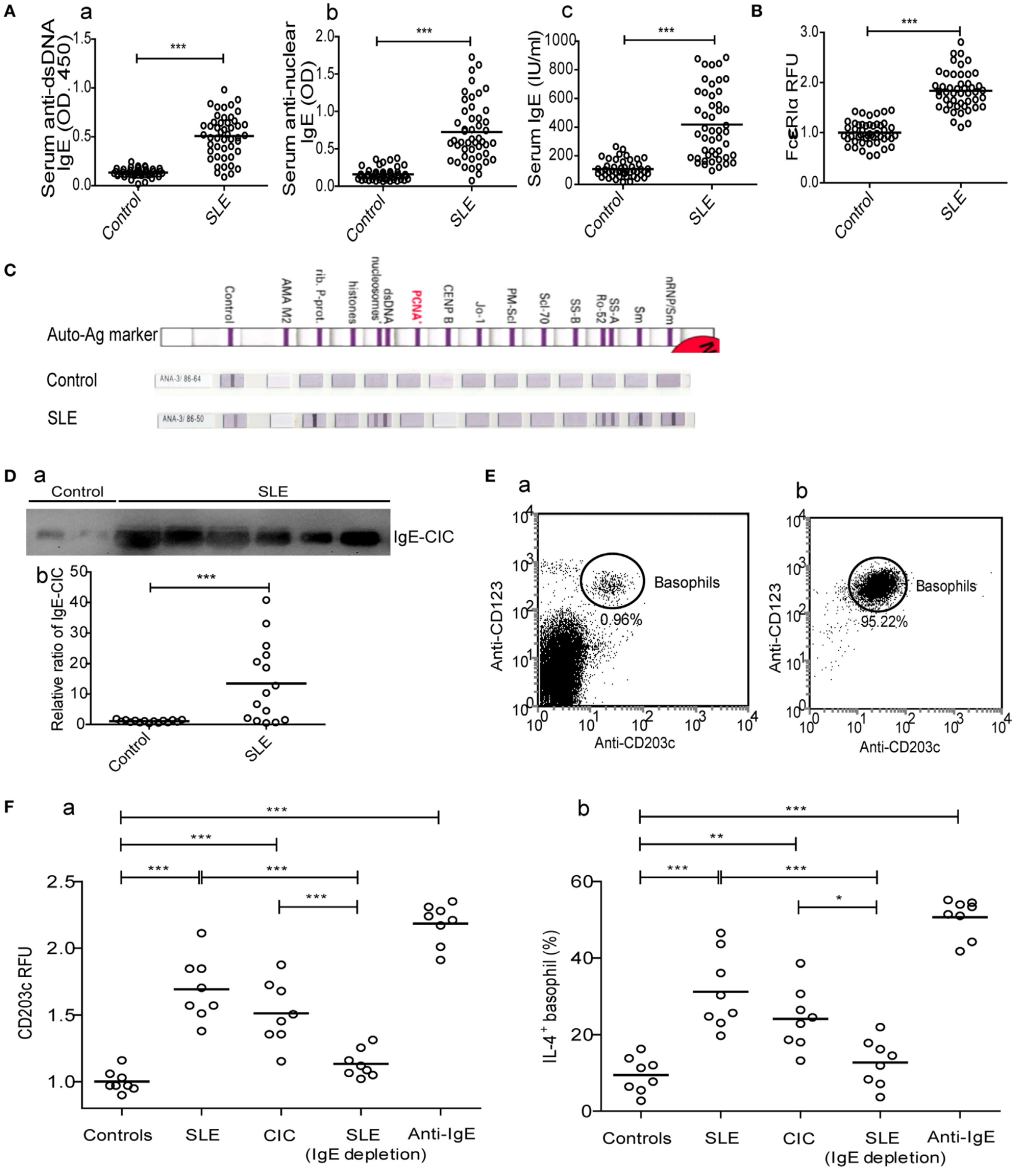
**Mechanism of peripheral basophil activation in patients with systemic lupus erythematosus (SLE)**. **(A)** Serum levels of anti-dsDNA IgE, antinuclear IgE, and total IgE (*n* = 48); **(B)** levels of FcɛRIα expression on peripheral basophils (*n* = 48); **(C)** antinuclear IgE profile (*n* = 36) (shown as representative images); and **(D)** the relative ratio of IgE-circulating immune complexes (CICs) (*n* = 15) in newly diagnosed patients with SLE and the same number of healthy controls. **(E)** Representative FCM dot-plot of peripheral basophils in PBMC of healthy controls (*n* = 6) (a) and post negatively isolated (b). **(F)** Activation [CD203c expression and interleukin (IL)-4 positive percentage] of basophils from healthy controls (*n* = 8) after stimulation with serum (20% of a total volume) of healthy control or newly diagnosed patients with SLE, CICs of newly diagnosed patients with SLE, serum (20% of a total volume) of newly diagnosed patients with SLE by depletion of IgE, or anti-IgE. *P < 0.05, **P < 0.01, ***P < 0.001. Data were analyzed *via* Student’s *t*-test **(A,B,D)** or one-way analysis of variance **(F)** and presented as scatter plots and are expressed as means.

Since peripheral basophils in patients with SLE were in an activated state, we next examined the mechanisms mediating their activation by looking into the potential involvement of IgE, a key inducer of basophil activation. The results showed that serum levels of anti-dsDNA IgE, antinuclear IgE and total IgE (Figure 2A), IgE-CIC (Figure 2D) in newly diagnosed patients with SLE were significantly higher than those in healthy controls, which was reconfirmed by the higher expression of the high-affinity receptor for IgE (FcεRIα) on basophils (Figure 2B). In addition, antinuclear IgE specific for auto-antigens, including nRNP/sm, Sm, SS-A, Ro-52, dsDNA, nucleosomes, rib-Prot, etc., can be detected in patients with SLE (*n* = 36) but not in healthy controls (*n* = 36) (Figure 2C). In an *in vitro* study, we found that peripheral basophils that were negatively isolated from healthy controls (Figure 2E) could be activated by the upregulation of CD203c expression (Figure 2F—a) and that the percentage of IL-4-positive cells (Figure 2F—b) could be increased by culturing with serum obtained from newly diagnosed patients with SLE, and IgE-CICs (Figure 2D) precipitated by PEG from the sera of newly diagnosed patients with SLE, but not by the serum of healthy controls or serum obtained from newly diagnosed patients with SLE by depletion of IgE. Basophils were activated with anti-IgE stimulation as a positive control (Figure 2F). These findings indicate that the presence of IgE, especially autoreactive IgE, mediate basophil activation in SLE.

### Basophils Promote Autoantibody Production by B Cells and Th17 Differentiation in SLE

**Figure 3:**
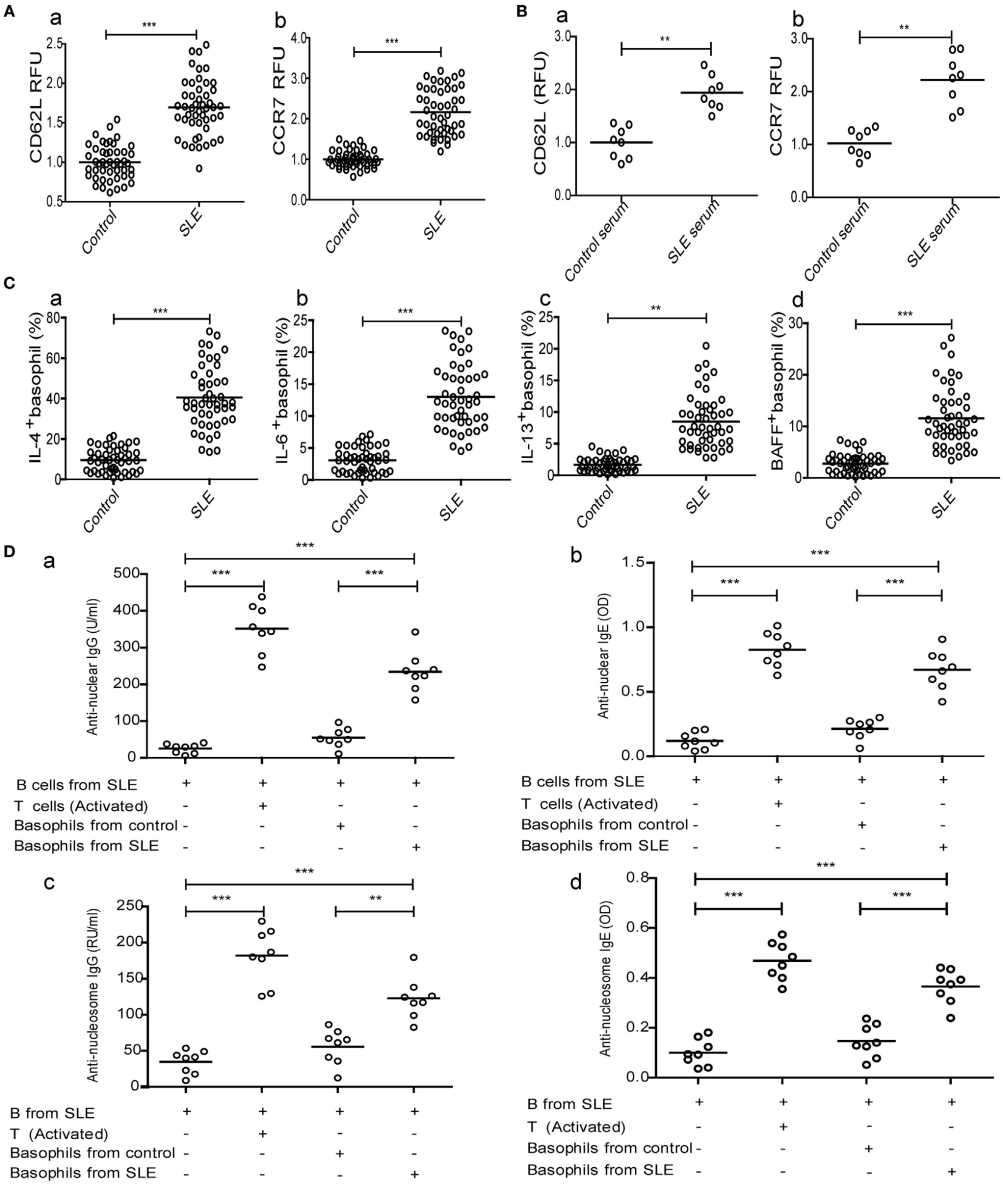

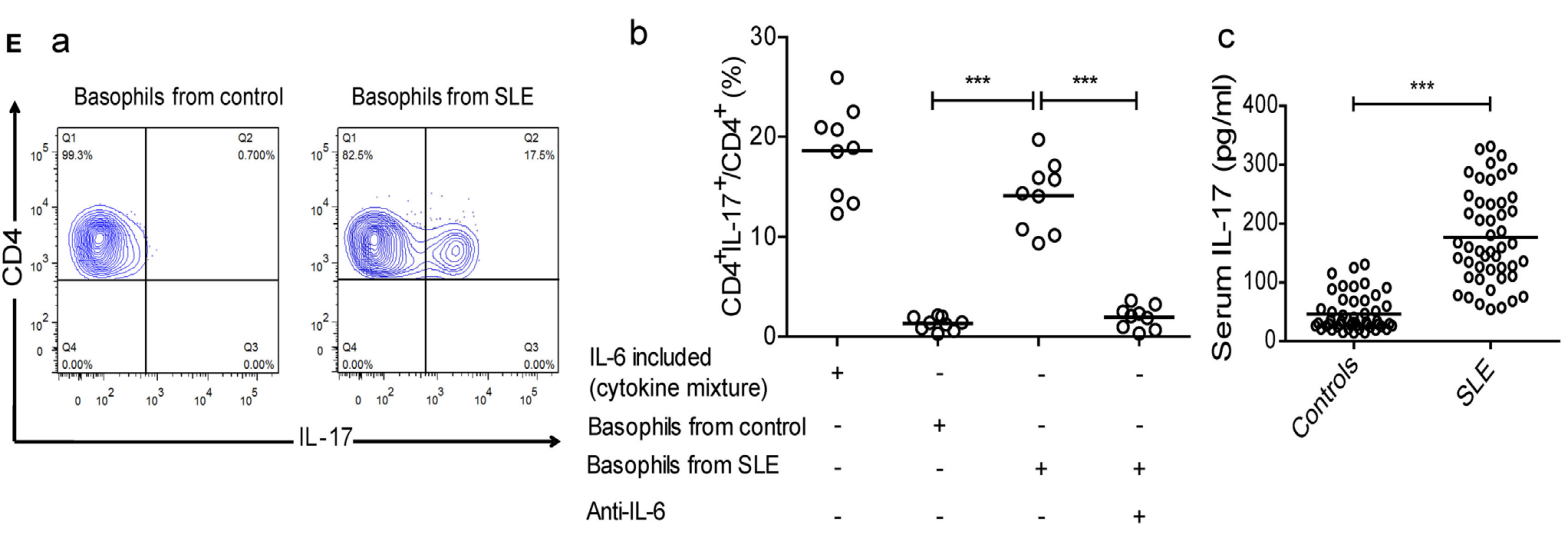
**Analysis of homing receptors expression on basophils and the effect of basophils on autoantibody production by B cells and Th17 differentiation in systemic lupus erythematosus (SLE)**. The expression of homing receptors (CD62L and CCR7) on **(A)** peripheral basophils of healthy controls (*n* = 48) and newly diagnosed patients with SLE (*n* = 48) and **(B)** basophils from healthy controls after stimulation with serum (20% of a total volume) of healthy control (*n* = 8) and newly diagnosed patients with SLE (*n* = 8) for 24 h. **(C)** The percentages of interleukin (IL)-4^−^ (a), IL-6^−^ (b), IL-13^−^ (c), and BAFF-positive (d) peripheral basophils in newly diagnosed patients with SLE and healthy controls (*n* = 48). **(D)** Antinuclear IgG (a), antinuclear IgE (b), anti-nucleosome IgG (c), and anti-nucleosome IgE (d) produced by SLE B cells post-coculturing with basophils from healthy controls or newly diagnosed patients with SLE and activated T cells for 12 days (*n* = 6). **(E)** The proportions of Th17 differentiation from SLE naïve CD4^+^ T cells post-coculturing in Th17 differentiation cytokines mixture (IL-6 included), or post-coculturing in Th17 differentiation cytokine mixture (excluding IL-6) with basophils from healthy controls (a, left, representative FCM plots), or basophils from newly diagnosed patients with SLE (a, right) in the absence or presence of anti-IL-6, respectively, and the statistical data (*n* = 9) (b), and serum levels of IL-17 in newly diagnosed patients with SLE (*n* = 48) and healthy controls (*n* = 48) (c). *P < 0.05, **P < 0.01, ***P < 0.001. Data were analyzed *via* Student’s *t*-test **(A–C**—e**)** or one-way analysis of variance **(D,E**—b**)** and presented as scatter plots and are expressed as means.

To clarify the triggers that decrease basophil counts in patients with SLE, we investigated the homing of basophils to the lymph nodes and the mechanisms that mediate this process. Our results showed that CD62L and CCR7, which are important for the homing of T cells to lymphoid tissues (37, 38), were upregulated on basophils obtained from newly diagnosed patients with SLE compared with those obtained from healthy controls (Figure 3A). Additionally, the expression levels of CD62L and CCR7 (Figure 3B) on peripheral basophils that were negatively isolated from healthy controls were increased by culturing with serum obtained from newly diagnosed patients with SLE but not by culturing with serum obtained from healthy controls.

We then examined the role of activated basophils from patients with SLE in autoantibody production by B cells from patients with SLE *in vitro*. The results showed that the percentages of IL-4-, IL-6-, IL-13-, and BAFF-positive peripheral basophils in newly diagnosed patients with SLE were significantly higher than those in healthy controls (Figure 3C). Moreover, basophils obtained from patients with SLE promoted antinuclear IgG and IgE production by B cells obtained from patients with SLE *in vitro*, but not those from healthy controls (Figure 3D—a and b). Also, anti-nucleosome IgG and IgE production has the same tendency (Figure 3D—c and d), while anti-tetanus IgG and IgE were not detected (data not shown). The coculture of activated T cells with B cells obtained from patients with SLE was used to produce autoantibodies as a positive control (Figure 3D). These findings demonstrate that basophils can amplify autoantibody production by B cells in SLE.

Next, we investigated the role of activated basophils from patients with SLE on Th17 differentiation from naïve CD4^+^ T cells from patients with SLE *in vitro*. Similar to what was observed with B cells, the results showed that isolated basophils from newly diagnosed patients with SLE significantly promoted Th17 differentiation compared to those obtained from healthy controls in the presence of a cytokine mixture (excluding IL-6) to induce Th17 differentiation (Figure 3E—a and b), and this effect could be suppressed by adding anti-IL-6 antibodies (Figure 3E—b), which was consistent with the finding that newly diagnosed patients with SLE had a higher percentage of IL-6-positive peripheral basophils (Figure 3C—b) and higher serum IL-17 levels (Figure 3E—c) than healthy controls. These findings demonstrate that basophils can promote Th17 differentiation in SLE.

### Activated Basophils Exacerbate Disease Progression in MRL-*lpr/lpr* Mice

Because clinical data and *in vitro* studies have indicated that activated basophils can promote autoantibody and IL-17 production and may exacerbate SLE, a lupus-prone MRL-*lpr/lpr* mice model that reflects the pathologies of human SLE (39) was employed to further investigate the role of basophils in SLE.

**Figure 4:**
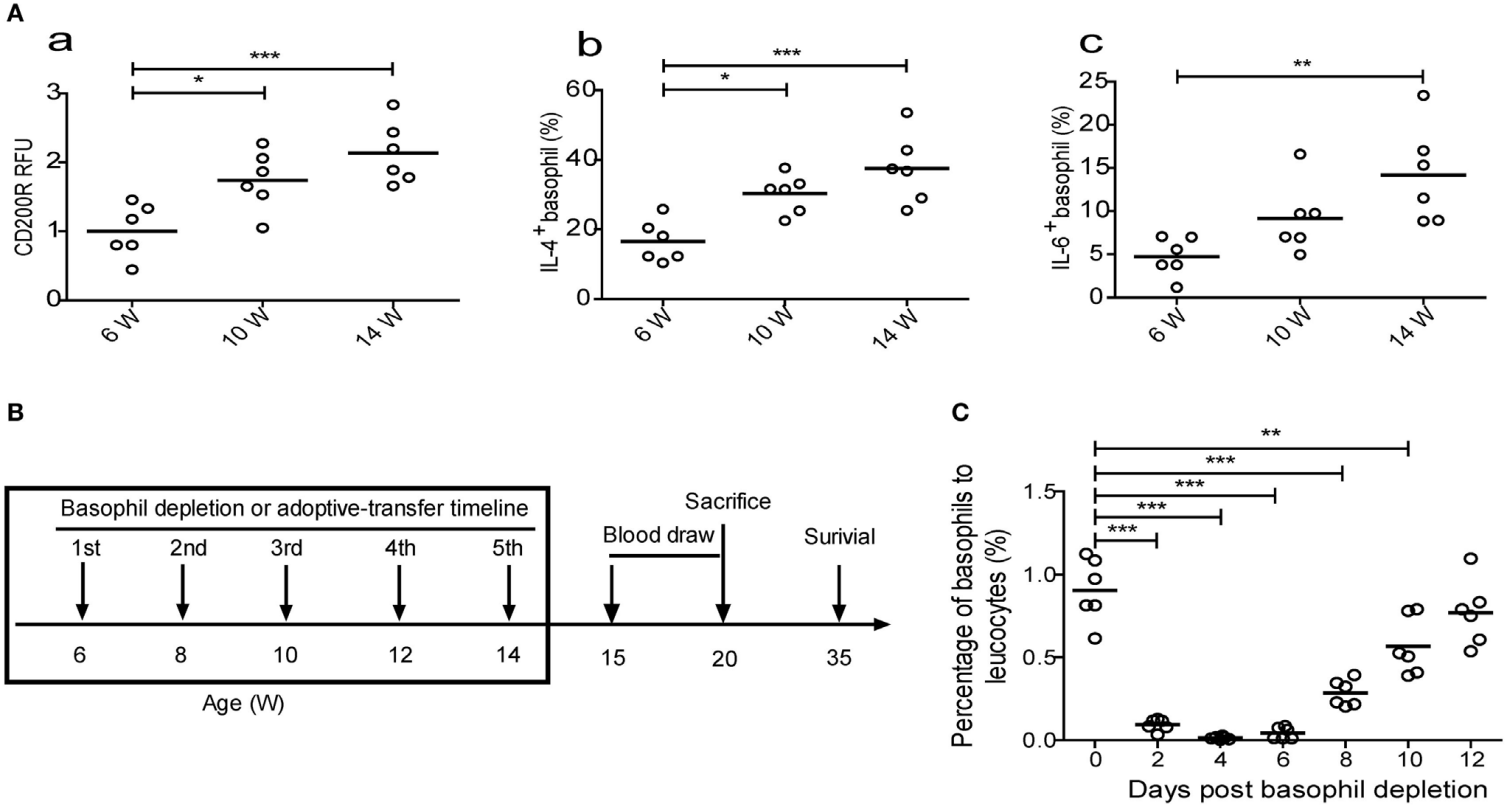

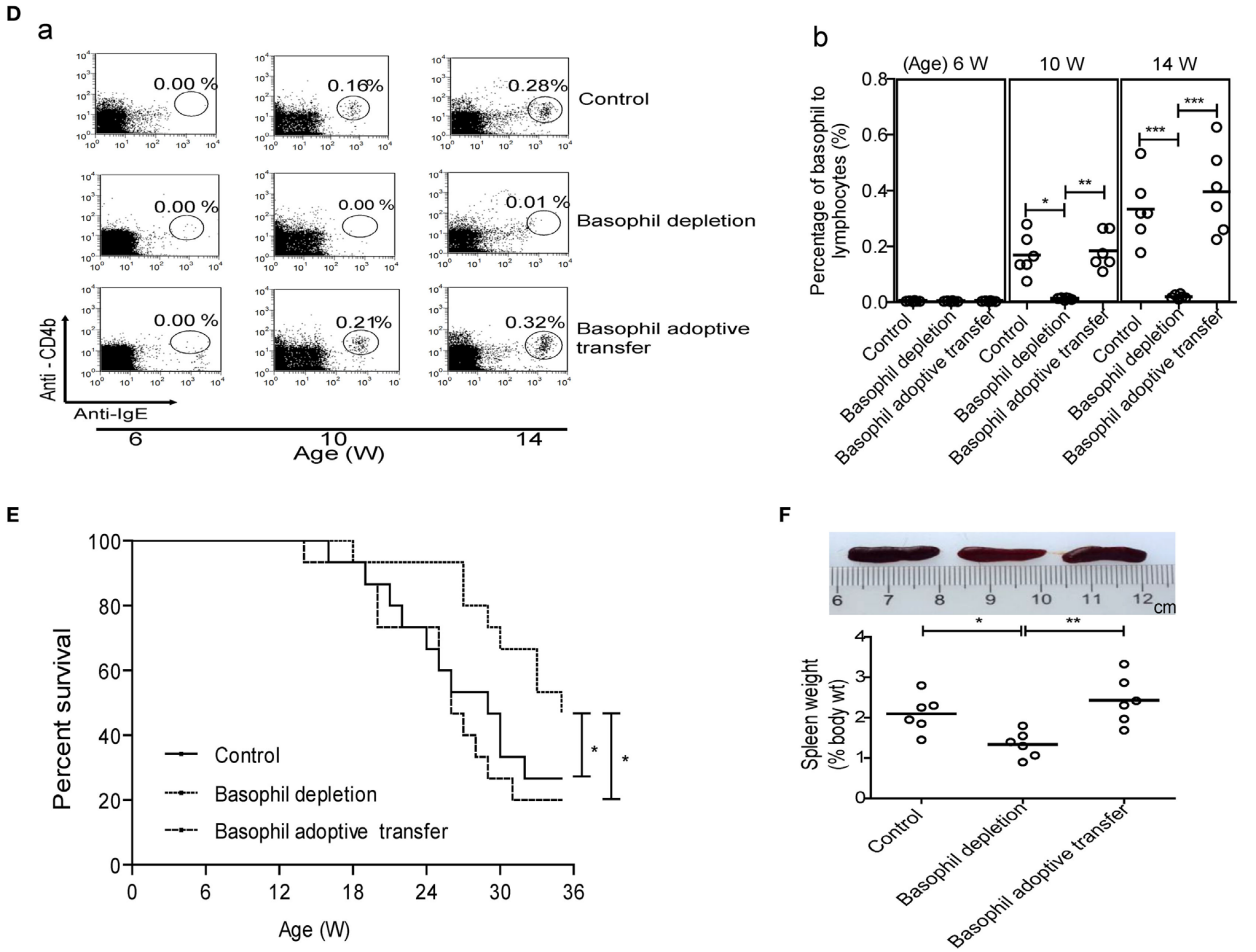
**Effect of basophil depletion or adoptive transfer on the survival of MRL-*lpr/lpr* mice**. **(A)** CD200R expression (a) on peripheral basophils and the percentages of interleukin (IL)-4^−^ (b) and IL-6-positive (c) peripheral basophils in MRL-*lpr/lpr* mice (*n* = 6). **(B)** Experimental design and time lines. **(C)** The changes of percentages of peripheral basophils to leukocytes in MRL-*lpr/lpr* mice (*n* = 6) after basophil depletion. **(D)** The changes of percentages of basophils to lymphocytes in the lymph nodes of control, basophil–depleted, and basophil-adoptive-transferred MRL-*lpr/lpr* mice (*n* = 6) (a) and statistical data (b). **(E)** The cumulative survival of three groups of MRL-*lpr/lpr* mice (*n* = 12) was monitored using the *Kaplan–Meier method*. **(F)** Representative images of the spleens of control, basophil–depleted, and basophil-adoptive-transferred MRL-*lpr/lpr* mice (from left to right) (a) and statistical data of the percentage of spleen weight to body weight of them (b) (*n* = 6). *P < 0.05, **P < 0.01, ***P < 0.001. Data were analyzed *via* one-way analysis of variance **(A,D,F)** and Student’s *t*-test **(C)** and presented as scatter plots and are expressed as means.

Compared to those in 6-week-old MRL-*lpr*/*lpr* mice, the expression levels of basophil activation markers, including CD200R (26), IL-4, and IL-6, were upregulated in 10- and 14-week-old mice (Figure 4A). Additionally, basophils were first detected in the lymph nodes of 10-week-old MRL-*lpr/lpr* mice and increased slightly in number as the mice aged (Figure 4D), a finding that is consistent with the upregulated expression of the homing receptors CD62L and CCR7 on basophils obtained from patients with SLE (Figure 3A).

We next evaluated the effects of basophil depletion and adoptive transfer on the development of autoimmune disorders in MRL-*lpr/lpr* mice. The experimental design and timelines was shown in Figure 4B. The results showed that one time of basophil depletion led to a significant decrease in the percentage of basophils in the peripheral blood (Figure 4C), lymph nodes (Figure 4D), and spleen (data not shown) of MRL-*lpr/lpr* mice and that this decrease lasted for more than 10 days. When basophils were depleted five times from 6 to 14 weeks of age, the survival of MRL-*lpr/lpr* mice was significantly prolonged compared with that of control and basophil-adoptive-transferred mice (Figure 4E).

**Figure 5:**
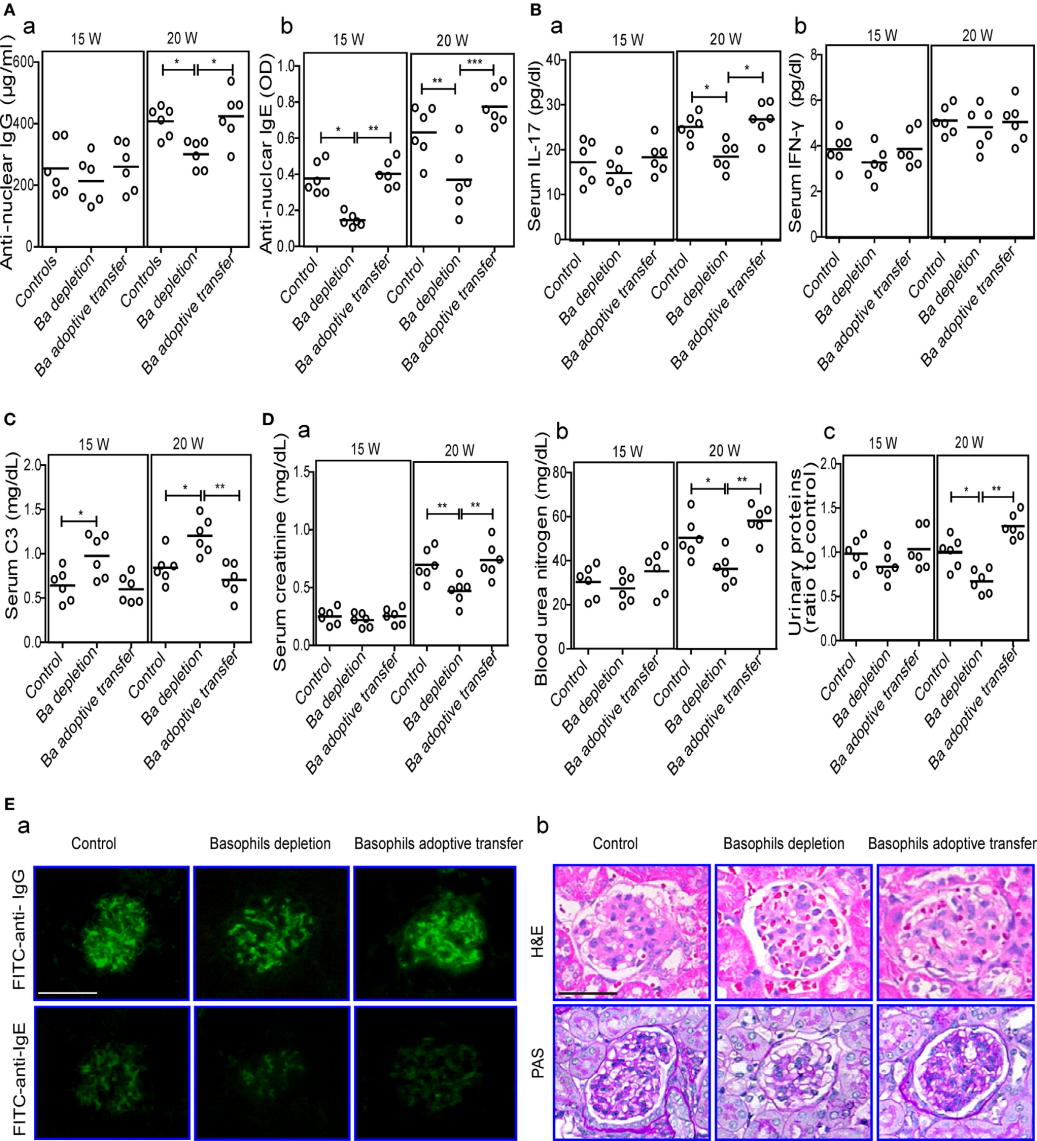
**Effects of basophil depletion or adoptive transfer on autoantibodies and inflammatory cytokines production and renal pathology of MRL-*lpr/lpr* mice**. Serum levels of antinuclear IgG and IgE **(A)**, interleukin (IL)-17 and IFN-γ **(B)**, C3 **(C)**, and **(D)** serum creatinine (a), blood urea nitrogen (b), and urinary proteins (c) in control, basophil–depleted, and basophil-adoptive-transferred MRL-*lpr/lpr* mice (*n* = 6). **(E)** Representative images of the fluorescence microscopy examination of IgG and IgE deposition **(E**—a**)**; and the light microscopy examination of the histopathology of glomerulonephritis using H&E and PAS staining **(E**—b**)** in control, basophil–depleted, and basophil-adoptive-transferred MRL-*lpr/lpr* mice (*n* = 6) at 20 weeks of age (scale bars, 50 µm). Ba, basophil. *P < 0.05, **P < 0.01, ***P < 0.001. Data were analyzed *via* one-way analysis of variance **(A–D)** and presented as scatter plots and are expressed as means.

In addition, basophil-depleted MRL-*lpr/lpr* mice exhibited a significant decrease in the ratio of spleen weight to body weight (Figure 4F) and the levels of serum antinuclear IgG and IgE at 20 weeks (Figure 5A), and an increase in the serum levels of C3 (Figure 5C) at 15 and 20 weeks compared with controls and basophil-adoptive-transferred mice. Basophil-depleted MRL-*lpr/lpr* mice exhibited significant decreases in the levels of serum IL-17, but not IFN-γ, at 20 weeks compared with control and basophil-adoptive-transferred mice (Figure 5B).

The influence of basophil depletion on renal injury in MRL-*lpr/lpr* mice was also assessed. Serum levels of creatinine, blood urea nitrogen, and urinary proteins were significantly decreased in basophil-depleted MRL-*lpr/lpr* mice at 20 weeks of age (Figure 5D). Basophil-depleted MRL-*lpr*/*lpr* mice exhibited decreased levels of IgG and IgE deposition in the glomeruli compared with the control and basophil-adoptive-transferred mice (Figure 5E—a). Additionally, the renal histopathology (including enlarged glomeruli, glomerular cell proliferation, and increased mesangial matrix) of basophil-depleted MRL-*lpr/lpr* mice was improved at 20 weeks compared with that of the controls (Figure 5E—b); however, basophil (from age-matched-MRL-*lpr/lpr* mice) adoptive-transferred MRL-*lpr/lpr* mice showed more severe glomerulonephritis than the controls (Figure 5E).

## Discussion

In this study, we used negatively isolated human basophils together with patient information and a lupus-prone mouse model to demonstrate that activated basophils amplify autoantibody and IL-17 production, thereby contributing to the pathogenesis of SLE.

Because basophils need to be activated to fulfill their biological functions (40, 41), we first demonstrated that basophils obtained from patients with SLE and MRL-*lpr/lpr* mice were activated due to higher expression of a series of activation markers and that some of these also correlated with disease activity in SLE. Then, we found that IgE, especially autoreactive IgE, mediated basophil activation in SLE, based on the detection of abundant total IgE, autoreactive IgE, and the higher expression of the high-affinity receptor for IgE (FcεRIα) on basophils in patients with SLE, and *in vitro* experiments including isolated basophils cocultured with the serum of patients with SLE and PEG precipitation CIC (containing IgE). The prevalence of autoreactive IgE in SLE was also observed by others (23, 42, 43).

In addition to activation, we simultaneously found that the numbers of peripheral basophils were decreased in patients with SLE. Thus, we evaluated where the basophils trafficked to following their activation. We found that basophils were initially detected in the lymph nodes in 10-week-old MRL-*lpr/lpr* mice, and this number increased slightly as the mice aged, which confirmed the results of a previous study showing that basophils were detected in the lymph nodes and spleen in two patients with SLE (23). These findings demonstrate the homing of basophils to lymph nodes in SLE. A subsequent study indicated that basophil migration to lymphoid tissues might mediated by CD62L and CCR7, based on the higher expression of these two homing receptors on basophils in patients with SLE as well as an *in vitro* experiment. Higher expression of CD62L by basophils of patients with SLE was also was also observed by others (23). Above all, homing to lymphoid tissues after basophil activation may contribute to decreased peripheral basophil numbers in SLE.

In this study, we mainly focused on the effects of activated basophils after homing to lymphoid tissues in SLE. The first key issue to clarify was the effect of activated basophils on autoantibody production by B cells in SLE. It has been confirmed that basophils can directly interact with B cells and provide helper signals to them through IL-4, IL-13, BAFF, and CD40L, driving their differentiation to antibody-producing cells (11). Our *in vitro* study showed that basophils from patients with SLE have the ability to promote autoantibodies including antinuclear and anti-nucleosome IgG and IgE production by B cells. For the immunological basis, this effect was possibly mediated by a synergistic effect of IL-4, IL-6, IL-13, and BAFF, together with the constitutive expression of CD40L (35, 44), as confirmed by our observation of a high expression level of these effectors by basophils of SLE patients (Figure 3C). This is consistent with the finding that basophil activation was correlated with the serum levels of antinuclear antibodies and with the deposition of immunoglobulins in the glomeruli of patients with SLE. Correspondingly, our *in vivo* study also showed that basophil-depleted MRL-*lpr/lpr* mice exhibited markedly decreased levels of serum autoantibodies and immunoglobulin deposition in glomeruli. However, anti-tetanus IgG/IgE in the culture supernatants was not detected (data not shown). For the next step, we plan to investigate which effectors play key roles in the synergistic effect of them in the context of SLE.

We next investigated another key issue: the effect of activated basophils on Th17 differentiation in SLE. Our *in vitro* study first showed that activated basophils obtained from patients with SLE could promote Th17 differentiation. Furthermore, basophil-depleted MRL-*lpr/lpr* mice exhibited significant decreases in their serum IL-17 levels. The finding that activated basophils enhance IL-17 production has also been reported in another autoimmune disease, inflammatory bowel disease (12). Further analysis showed that IL-6, which is also defined as a Th17-inducing cytokine (20) due to its role in the regulation of Th17 differentiation (45), was necessary for promoting Th17 differentiation and IL-17 production by activated basophils in patients with SLE, as further confirmed by our observation that basophils obtained from patients with SLE and aged MRL-*lpr/lpr* mice both exhibited a higher expression of IL-6.

Moreover, to directly verify the role of basophils in SLE progression, a selective basophil-depletion murine model was successfully established, as reported previously (18, 46). Our results showed that basophils were activated along with aging, and the homing of basophils to lymph nodes was significantly inhibited after depletion of basophils in MRL-*lpr/lpr* mice. Consequently, the basophil-depleted mice exhibited benefits on renal function and attenuated the progression of glomerulonephritis, finally led to the extension of life span, which was accompanied with significant decreases in autoantibody and IL-17 production. However, basophil-adoptive-transferred MRL-*lpr/lpr* mice exhibited the opposite tendency, although the results were not significant possibly because the peripheral basophils were sufficient for migration, which is consistent with the observation that basophils were not significantly increased in the lymph nodes of the basophil-adoptive-transferred mice. In addition, we also used the antibody anti-CD200R3 (Ba103; Hycult, Uden, The Netherlands) for basophil depletion (47), and the results showed the same tendency (data not shown). In another study, basophils were found to be necessary for the development of autoimmune disease in Lyn-deficient mice (23). We noted that due to the induction of antibodies to basophil-depletion antibodies, the prolonged use of them *in vivo* was limited, and also the efficiency of basophil depletion in MRL-*lpr/lpr* mice was decreased for the fifth basophil depletion compared with the first time even it can led to a significant decrease in the percentage of basophils in the peripheral blood lasted for more than 6 days. Recently, several kinds of basophil ablation mouse models, Tg mouse, have been generated (48, 49), which will help us to further study the critical roles of basophils in SLE in the future. These findings demonstrate that activated basophils exacerbate disease progression in MRL-*lpr/lpr* mice.

## Conclusion

**Figure 6:**
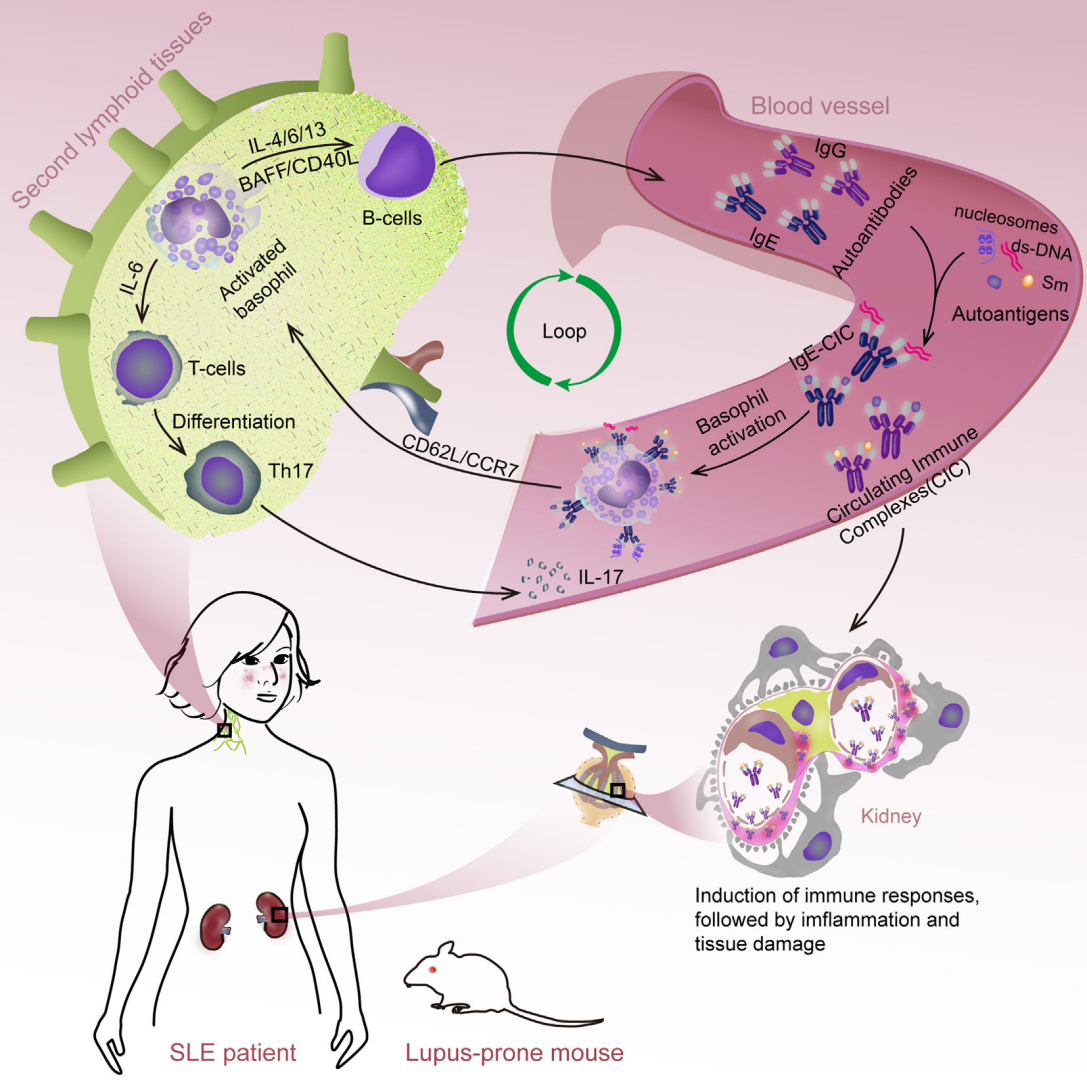
**Schematic representation of systemic lupus erythematosus (SLE) exacerbation by basophil activation-dependent autoantibody and interleukin (IL)-17 production**. During the progress of SLE, peripheral basophils could migrate to secondary lymphoid tissues in response to the increased expression of CD62L/CCR7 post their activation mediated by (autoreactive) IgE. At these sites, basophils could exacerbate SLE by promoting autoantibody production possibly mediated by a synergistic effect of IL-4, IL-6, IL-13, BAFF, and CD40L, and Th17 differentiation by IL-6 resulted in the exacerbating of disease.

This study demonstrates that basophils can exacerbate SLE through activation-dependent autoantibody and IL-17 production (Figure 6). These findings provide novel insights into the pathogenesis of SLE and may lead to the development of new therapeutic strategies for treating SLE.

## Author Contributions

QP and H-fL conceived and designed the experiments. QP, LG, HX, YF, LL, ZD, and LingY performed the experiments. QP and H-fL analyzed the data. YF, LL, ZD, LingY, NA, and CY contributed reagents/materials/analysis tools. QP, JZ, CD, LinY, and H-fL contributed to the writing of the manuscript. All the authors reviewed and approved the final manuscript.

## Conflict of Interest Statement

The authors declare that this study was conducted in the absence of any commercial or financial relationships that could be construed as a potential conflict of interest.

## Supplementary Material

Figure S1 **Representative data of FACS for basophil frequency, CD203c, and CD63^+^ basophil**. Gate 1 (①) isolates the peripheral leukocytes, and gate 2 (②) detects the low side-scatter (SSC), FcεRIα^+^ population of cells. Then, with a double gating strategy, gate 3 (③) detects CD123^+^CD203c^+^ basophil population. For CD63^+^ basophil, a followed gate 4 (④) detects the low SSC, FcεRIα^+^CD123^+^CD203c^+^CD63^+^ population of basophil. Gates were set according to isotype controls antibodies, respectively. The results were analyzed with FlowJo Software in a pseudocolor dot-plot.

Figure S2 **Numbers and activation of peripheral basophils and their correlations with disease activity and autoantibody production in patients with systemic lupus erythematosus (SLE) treated with *Tripterygium wilfordi***. **(A)** Correlations between the activation (CD203c expression) of peripheral basophils and disease activity [SLE disease activity index (SLEDAI) score] (a), and levels of their serum antinuclear IgG (b) or IgE (c) of patients with SLE treated with *T. wilfordi* (*n* = 54) were analyzed. **(B)** Changes in the SLEDAI score (d), the numbers (e), and activation (CD203c expression) (f) of peripheral basophils in patients with SLE treated with *T. wilfordi* (*n* = 4) who were followed up for 3 months posttreatment. **P* < 0.05, ***P* < 0.01, ****P* < 0.001. Data were analyzed *via* Spearman’s rank correlation **(A)** or one-way analysis of variance **(B)** and presented as scatter plots and are expressed as means.
