# Supplementary figures and images for: Basophil Activation-Dependent Autoantibody and Interleukin-17 Production Exacerbate Systemic Lupus Erythematosus
Source: Front Immunol. 2017 Mar 27;8:348. doi: 10.3389/fimmu.2017.00348 (PMC5366357; doi:10.3389/fimmu.2017.00348)

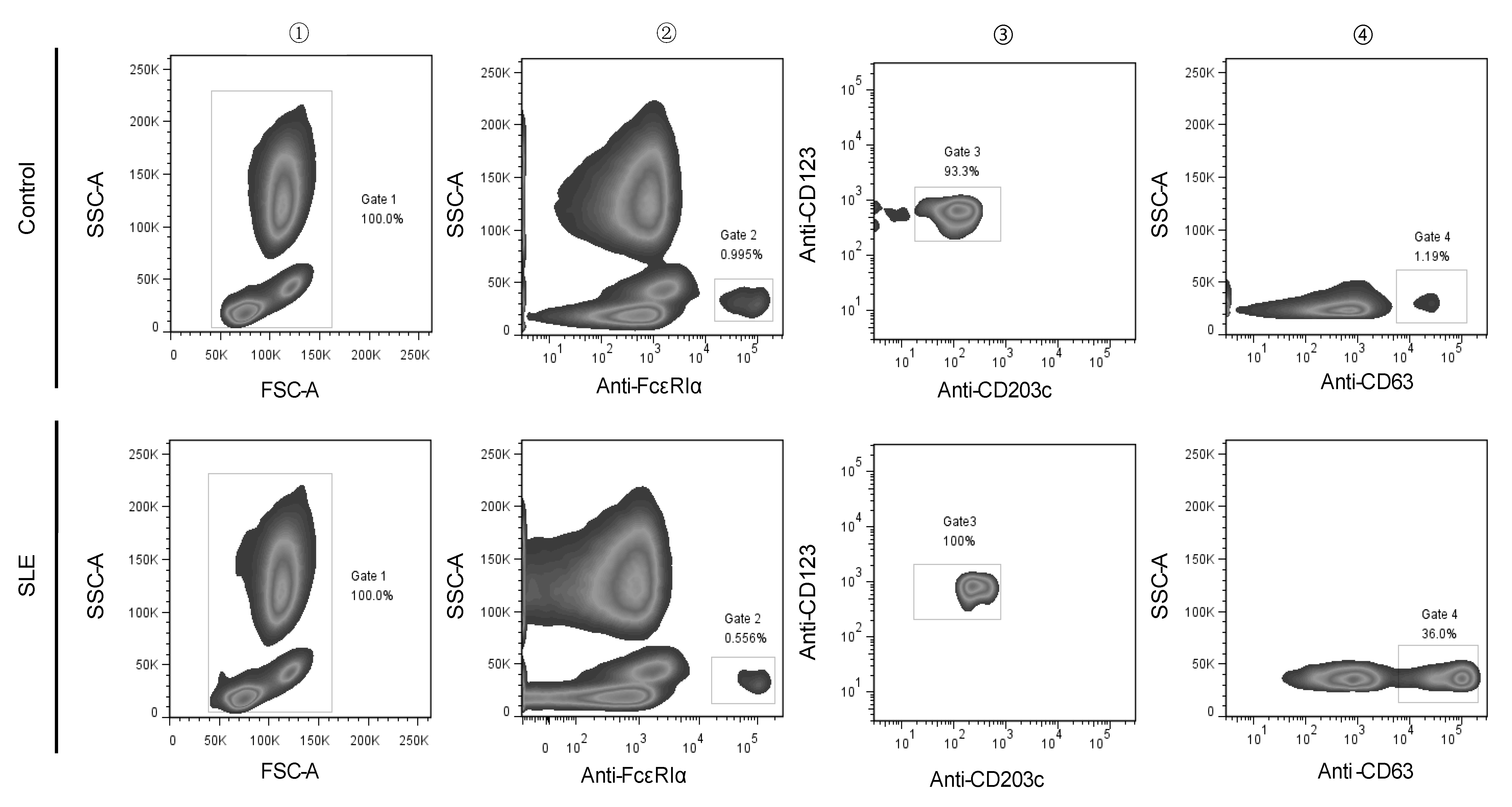

Supplement: Figure S1 — Representative data of FACS for basophil frequency, CD203c, and CD63+ basophil. Gate 1 (①) isolates the peripheral leukocytes, and gate 2 (②) detects the low side-scatter (SSC), FcεRIα+ population of cells. Then, with a double gating strategy, gate 3 (③) detects CD123+CD203c+ basophil population. For CD63+ basophil, a followed gate 4 (④) detects the low SSC, FcεRIα+CD123+CD203c+CD63+ population of basophil. Gates were set according to isotype controls antibodies, respectively. The results were analyzed with FlowJo Software in a pseudocolor dot-plot. [file Image_1.tiff]

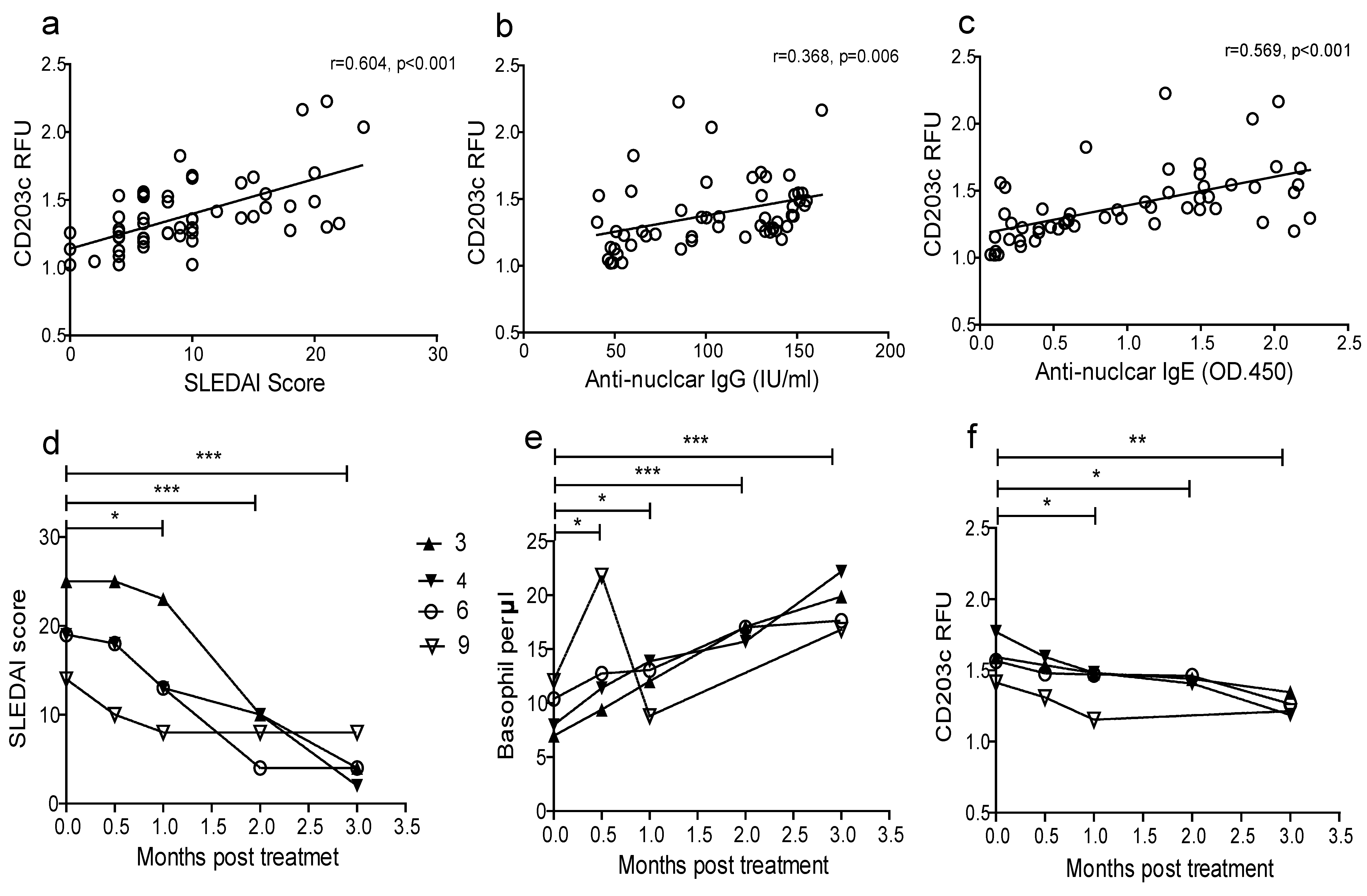

Supplement: Figure S2 — Numbers and activation of peripheral basophils and their correlations with disease activity and autoantibody production in patients with systemic lupus erythematosus (SLE) treated with Tripterygium wilfordi. (A) Correlations between the activation (CD203c expression) of peripheral basophils and disease activity [SLE disease activity index (SLEDAI) score] (a), and levels of their serum antinuclear IgG (b) or IgE (c) of patients with SLE treated with T. wilfordi (n = 54) were analyzed. (B) Changes in the SLEDAI score (d), the numbers (e), and activation (CD203c expression) (f) of peripheral basophils in patients with SLE treated with T. wilfordi (n = 4) who were followed up for 3 months posttreatment. *P < 0.05, **P < 0.01, ***P < 0.001. Data were analyzed via Spearman’s rank correlation (A) or one-way analysis of variance (B) and presented as scatter plots and are expressed as means. [file Image_2.tiff]
